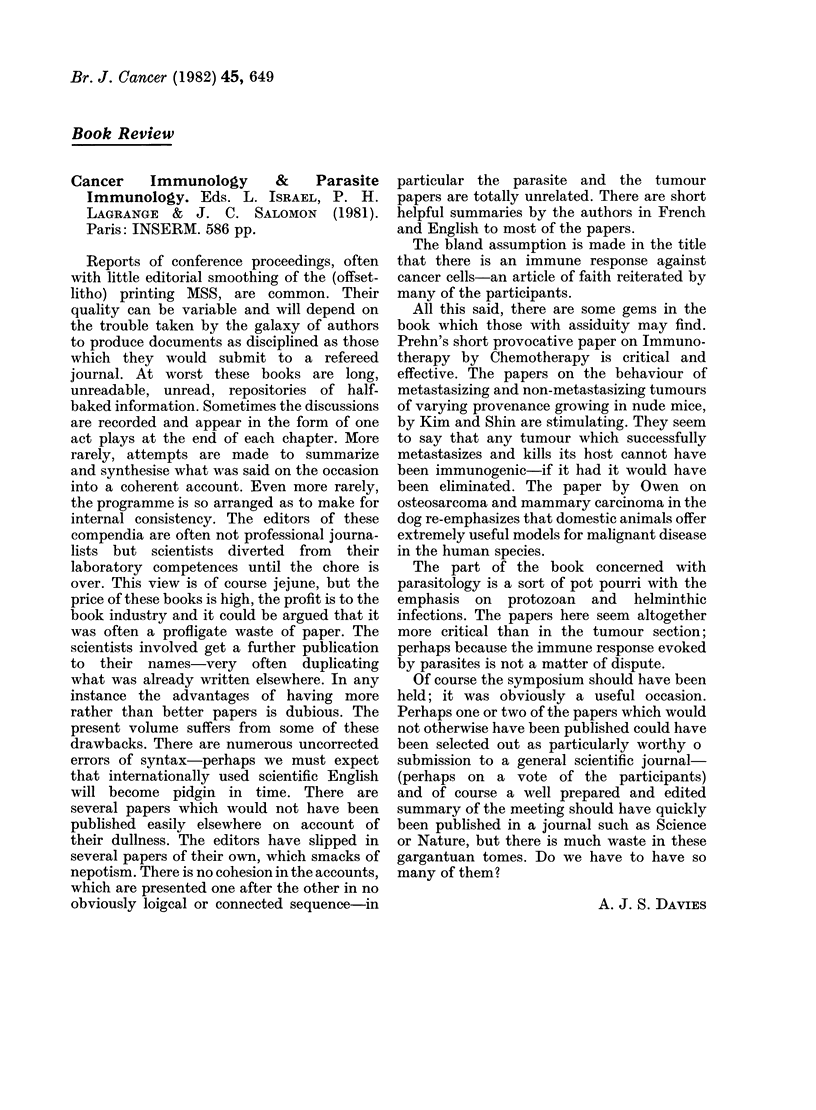# Cancer Immunology & Parasite Immunology

**Published:** 1982-04

**Authors:** A. J. S. Davies


*Br. J. Cancer* (1982) **45**, 649

### *Book Review*

**Cancer Immunology & Parasite Immunology.** Eds. L. Israel, P. H. Lagrange & J. C. Salomon (1981). Paris: INSERM. 586 pp.

Reports of conference proceedings, often with little editorial smoothing of the (offset-litho) printing MSS, are common. Their quality can be variable and will depend on the trouble taken by the galaxy of authors to produce documents as disciplined as those which they would submit to a refereed journal. At worst these books are long, unreadable, unread, repositories of half-baked information. Sometimes the discussions are recorded and appear in the form of one act plays at the end of each chapter. More rarely, attempts are made to summarize and synthesise what was said on the occasion into a coherent account. Even more rarely, the programme is so arranged as to make for internal consistency. The editors of these compendia are often not professional journalists but scientists diverted from their laboratory competences until the chore is over. This view is of course jejune, but the price of these books is high, the profit is to the book industry and it could be argued that it was often a profligate waste of paper. The scientists involved get a further publication to their names—very often duplicating what was already written elsewhere. In any instance the advantages of having more rather than better papers is dubious. The present volume suffers from some of these drawbacks. There are numerous uncorrected errors of syntax—perhaps we must expect that internationally used scientific English will become pidgin in time. There are several papers which would not have been published easily elsewhere on account of their dullness. The editors have slipped in several papers of their own, which smacks of nepotism. There is no cohesion in the accounts, which are presented one after the other in no obviously loigcal or connected sequence—in particular the parasite and the tumour papers are totally unrelated. There are short helpful summaries by the authors in French and English to most of the papers.

The bland assumption is made in the title that there is an immune response against cancer cells—an article of faith reiterated by many of the participants.

All this said, there are some gems in the book which those with assiduity may find. Prehn's short provocative paper on Immunotherapy by Chemotherapy is critical and effective. The papers on the behaviour of metastasizing and non-metastasizing tumours of varying provenance growing in nude mice, by Kim and Shin are stimulating. They seem to say that any tumour which successfully metastasizes and kills its host cannot have been immunogenic—if it had it would have been eliminated. The paper by Owen on osteosarcoma and mammary carcinoma in the dog re-emphasizes that domestic animals offer extremely useful models for malignant disease in the human species.

The part of the book concerned with parasitology is a sort of pot pourri with the emphasis on protozoan and helminthic infections. The papers here seem altogether more critical than in the tumour section; perhaps because the immune response evoked by parasites is not a matter of dispute.

Of course the symposium should have been held; it was obviously a useful occasion. Perhaps one or two of the papers which would not otherwise have been published could have been selected out as particularly worthy o submission to a general scientific journal—(perhaps on a vote of the participants) and of course a well prepared and edited summary of the meeting should have quickly been published in a journal such as Science or Nature, but there is much waste in these gargantuan tomes. Do we have to have so many of them?

A. J. S. Davies